# 4-(Pyrimidin-2-yl)piperazin-1-ium (*E*)-3-carb­oxy­prop-2-enoate

**DOI:** 10.1107/S1600536814011489

**Published:** 2014-05-24

**Authors:** Thammarse S. Yamuna, Manpreet Kaur, Jerry P. Jasinski, H. S. Yathirajan

**Affiliations:** a Department of Studies in Chemistry, University of Mysore, Manasagangotri, Mysore 570 006, India; b Department of Chemistry, Keene State College, 229 Main Street, Keene, NH 03435-2001, USA

## Abstract

In the cation of the title salt, C_8_H_13_N_4_^+^·C_4_H_3_O_4_^−^, the piperazinium ring adopts a slightly distorteded chair conformation. In the crystal, a single strong O—H⋯O inter­molecular hydrogen bond links the anions, forming chains along the *c-*axis direction. The chains of anions are linked by the cations, *via* N—H⋯O hydrogen bonds, forming sheets parallel to (100). These layers are linked by weak C—H⋯O hydrogen bonds, forming a three-dimensional structure. In addition, there are weak π–π inter­actions [centroid–centroid distance = 3.820 (9) Å] present involving inversion-related pyrimidine rings.

## Related literature   

For heterocyclic compounds that exhibit a broad spectrum of biological activities see: Amin *et al.* (2009 ▶); Clark *et al.* (2007 ▶); Ibrahim & El-Metwally (2010 ▶); Kim *et al.* (2010 ▶); Kuyper *et al.* (1996 ▶); Padmaja *et al.* (2009 ▶); Pandey *et al.* (2004 ▶). For piperazine-based compounds of biological and chemotherapeutic importance, see: Abdel-Jalil *et al.* (2010 ▶). For piperazine derivatives that have reached the stage of clinical application among the known drugs to treat anxiety, see: Tollefson *et al.* (1991 ▶). For related structures, see: Betz *et al.* (2011 ▶); Fun *et al.* (2012 ▶); Jasinski *et al.* (2010 ▶, 2011 ▶); Kavitha *et al.* (2013 ▶); Ravikumar & Sridhar (2005 ▶); Siddegowda *et al.* (2011 ▶). For puckering parameters, see Cremer & Pople (1975 ▶). For standard bond lengths, see: Allen *et al.* (1987 ▶).
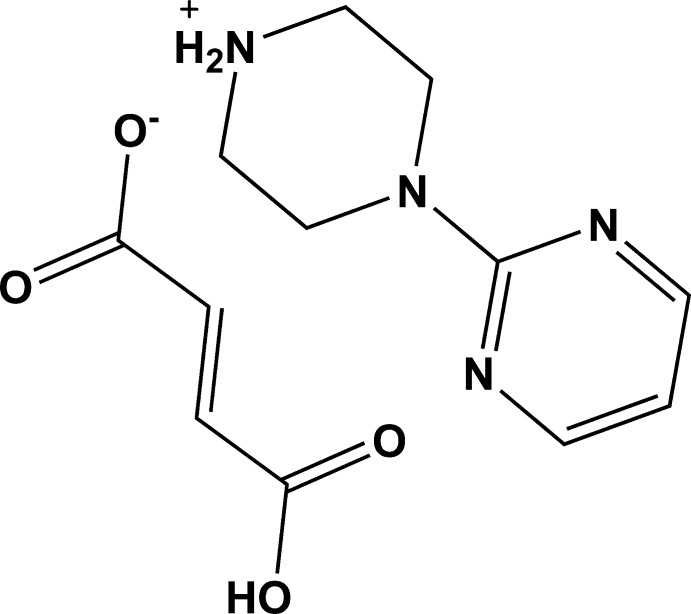


## Experimental   

### 

#### Crystal data  

C_8_H_13_N_4_^+^·C_4_H_3_O_4_^−^
*M* *_r_* = 280.29
Monoclinic, 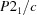

*a* = 12.3425 (5) Å
*b* = 7.0365 (3) Å
*c* = 14.7178 (6) Å
β = 94.213 (3)°
*V* = 1274.77 (9) Å^3^
*Z* = 4
Cu *K*α radiation
μ = 0.94 mm^−1^
*T* = 173 K
0.22 × 0.16 × 0.06 mm

#### Data collection  

Agilent Xcalibur Eos Gemini diffractometer
Absorption correction: multi-scan (*CrysAlis PRO* and *CrysAlis RED*; Agilent, 2012 ▶). *T*_min_ = 0.840, *T*_max_ = 1.000
8262 measured reflections
2455 independent reflections
2090 reflections with *I* > 2σ(*I*)
*R*_int_ = 0.039

#### Refinement  

*R*[*F* ^2^ > 2σ(*F* ^2^)] = 0.040
*wR*(*F* ^2^) = 0.111
*S* = 1.06
2455 reflections
186 parameters
H atoms treated by a mixture of independent and constrained refinement
Δρ_max_ = 0.23 e Å^−3^
Δρ_min_ = −0.27 e Å^−3^

### 

Data collection: *CrysAlis PRO* (Agilent, 2012 ▶); cell refinement: *CrysAlis PRO*; data reduction: *CrysAlis RED* (Agilent, 2012 ▶); program(s) used to solve structure: *SUPERFLIP* (Palatinus & Chapuis, 2007 ▶); program(s) used to refine structure: *SHELXL2012* (Sheldrick, 2008 ▶); molecular graphics: *OLEX2* (Dolomanov *et al.*, 2009 ▶); software used to prepare material for publication: *OLEX2*.

## Supplementary Material

Crystal structure: contains datablock(s) I. DOI: 10.1107/S1600536814011489/su2736sup1.cif

Structure factors: contains datablock(s) I. DOI: 10.1107/S1600536814011489/su2736Isup2.hkl

Supporting information file. DOI: 10.1107/S1600536814011489/su2736Isup3.cml

CCDC reference: 1003662

Additional supporting information:  crystallographic information; 3D view; checkCIF report

## Figures and Tables

**Table 1:** Hydrogen-bond geometry (Å, °)

*D*—H⋯*A*	*D*—H	H⋯*A*	*D*⋯*A*	*D*—H⋯*A*
N1*A*—H1*AA*⋯O2*B* ^i^	0.99	1.79	2.7601 (15)	166
N1*A*—H1*AB*⋯O4*B* ^ii^	0.99	1.78	2.7493 (16)	167
C8*A*—H8*A*⋯O2*B* ^iii^	0.95	2.53	3.3133 (18)	140
O1*B*—H1*B*⋯O3*B* ^iv^	1.12 (3)	1.35 (3)	2.4679 (13)	176 (3)

Symmetry codes: (i) 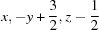; (ii) 
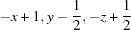
; (iii) 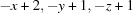; (iv) 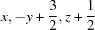.

**Fig. 1.**
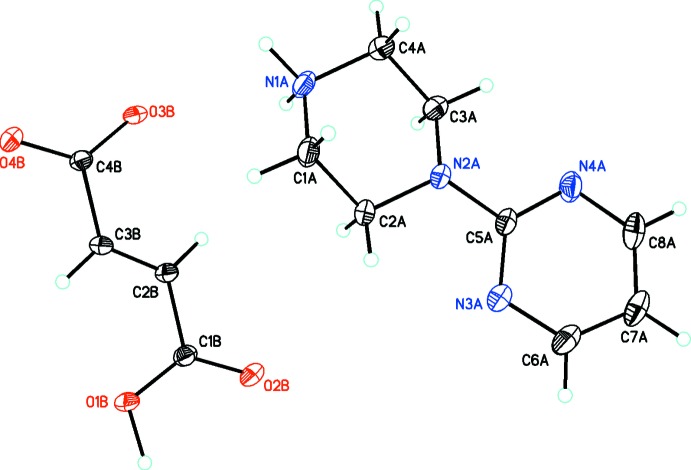
A view of the molecular structure of the title salt, with atom labelling. Displacement ellipsoids are drawn at the 30% probability level.

**Fig. 2.**
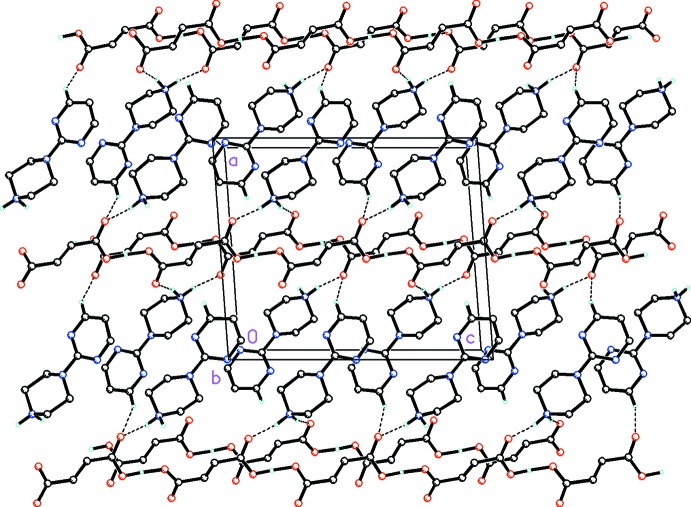
A view along the b axis of the crystal packing of the title salt. Hydrogen bonds are shown as dashed lines (see Table 1 for details; H atoms not involved in hydrogen bonding have been omitted for clarity).

## supplementary crystallographic information

### 1. Comment

Pyrimidine derivatives have attracted organic chemists due to their biological and chemotherapeutic importance. Related fused heterocycles are important classes of heterocyclic compounds that exhibit a broad spectrum of biological activities such as anticancer (Amin *et al.*, 2009; Pandey *et al.*, 2004), antiviral (Ibrahim & El-Metwally, 2010), antibacterial (Kuyper *et al.*, 1996), antioxidant (Padmaja *et al.* , 2009), antidepressant (Kim *et al.*, 2010) and anti-inflammatory (Clark *et al.*, 2007). Piperazine-based compounds have been employed as antibacterial, antidepressant, and antitumor drugs, and as α adrenoceptor antagonists, CCR5 receptor antagonists, 5-HT7 receptor antagonists, and adenosine A2a receptor antagonists (Abdel-Jalil *et al.*, 2010). Several piperazine derivatives have reached the stage of clinical application among the known drugs that are used to treat anxiety including the pyrimidinyl piperazinyl compounds, buspirone and BuSpar (Tollefson *et al.*, 1991). The incorporation of two moieties increases biological activity of both the molecules. Our research group has published many papers on incorporated heterocyclic ring structures, viz; imatinibium dipicrate [systematic name: 1-methyl-4-(4-{4-methyl-3- [4-(3-pyridyl)pyrimidin-2-ylamino]anilinocarbonyl}benzyl)piperazine-1,4- diium dipicrate, (Jasinski *et al.*, 2010), 1-(2-hydroxyethyl)-4-{3-[(E)-2-(trifluoromethyl)-9H-thioxanthen-9- ylidene]propyl}piperazine-1,4-diium bis(3-carboxyprop-2-enoate) (Siddegowda *et al.*, 2011), lomefloxacinium picrate (Jasinski *et al.*, 2011), paliperidone: 3-{2-[4-(6-fluoro-1,2-benzoxazol-3- yl)piperidin-1-yl]ethyl}-9-hydroxy-2-methyl-1,6,7,8,9,9a-hexahydropyrido [1,2-a]pyrimidin-4-one (Betz *et al.*, 2011), 4-[3,5-bis(2-hydroxy phenyl)-1H-1,2,4-triazol-1-yl]benzoic acid dimethylformamide monosolvate (Fun *et al.*, 2012), and other related crystal structures are quetiapine hemifumarate (systematic name: 1-[2-(2-hydroxyethoxy) ethyl]- 4-(dibenzo[b,f][1,4]thiazepin-11-yl)piperazinium hemifumarate (Ravikumar & Sridhar, 2005), Cinnarizinium fumarate (Kavitha *et al.*, 2013). In view of the importance of the incorporated of heterocyclic ring compounds and derivative of pyrimidyl piperazines, this paper reports the crystal structure of the title salt.

The title salt crystallizes with one independent monoprotonated piperazinium cation (A) and one independent fumarate anion (B) in the asymmetric unit (Fig. 1). In the cation, the piperazinium ring adopts a slightly distorted chair conformation (puckering parameters Q, θ, and φ = 0.5738 (14) Å, 5.20 (14)° and 21.1 (16)° (Cremer & Pople, 1975). Bond lengths are in normal ranges (Allen *et al.*, 1987).

In the crystal, a single strong short O1B—H1B···O3B hydrogen bond links the anions resulting in chains along the *c* axis (Table 1 and Fig. 2). The chains are linked via N—H···O hydrogen bonds to form sheets parallel to (100). A weak C8A—H8A···O2B hydrogen bond links the cations and anions forming a three-dimensional structure with alternate layers of cations and anions (Table 1 and Fig. 2). In addition, weak π–π interactions involving inversion related pyrimidine rings are present [Cg–Cg^i^ = 3.820 (9) Å; symmetry code:(i) -x+2, -y+1, -z+1; Cg is the centroid of the pyrimidine ring N3A/N4A/C5A-C8A].

### 2. Experimental

1-(2-Pyrimidyl)piperazine (Sigma-Aldrich; 0.2 g, 1.2179 mmol) and fumaric acid (0.1412 g, 1.2179 mmol ) were dissolved in 10 ml of dimethylsulfoxide and stirred at 333 K for 20 minutes. After a few days, colourless block-like crystals were obtained on slow evaporation of the solvent [M.p: 433-438 K].

### 3. Refinement

Atom H1B was freely refined and all of the remaining H atoms were placed in their calculated positions and refined using the riding model approach: N-H = 0.99 Å for NH_2_ H atoms, C—H = 0.95 and 0.99Å for CH and CH_2_ H atoms, respectively, with U_iso_(H) = 1.2U_eq_(N,C).

### Crystal data

**Table:**

C_8_H_13_N_4_^+^·C_4_H_3_O_4_^−^	*F*(000) = 592
*M**_r_* = 280.29	*D*_x_ = 1.460 Mg m^−^^3^
Monoclinic, *P*2_1_/*c*	Cu *K*α radiation, λ = 1.54184 Å
*a* = 12.3425 (5) Å	Cell parameters from 3297 reflections
*b* = 7.0365 (3) Å	θ = 3.6–71.5°
*c* = 14.7178 (6) Å	µ = 0.94 mm^−^^1^
β = 94.213 (3)°	*T* = 173 K
*V* = 1274.77 (9) Å^3^	Block, colourless
*Z* = 4	0.22 × 0.16 × 0.06 mm

### Data collection

**Table:**

Agilent Xcalibur Eos Gemini diffractometer	2455 independent reflections
Radiation source: Enhance (Cu) X-ray Source	2090 reflections with *I* > 2σ(*I*)
Detector resolution: 16.0416 pixels mm^-1^	*R*_int_ = 0.039
ω scans	θ_max_ = 71.1°, θ_min_ = 3.6°
Absorption correction: multi-scan (*CrysAlis PRO* and *CrysAlis RED*; Agilent, 2012).	*h* = −14→15
*T*_min_ = 0.840, *T*_max_ = 1.000	*k* = −8→6
8262 measured reflections	*l* = −17→17

### Refinement

**Table:**

Refinement on *F*^2^	Hydrogen site location: mixed
Least-squares matrix: full	H atoms treated by a mixture of independent and constrained refinement
*R*[*F*^2^ > 2σ(*F*^2^)] = 0.040	*w* = 1/[σ^2^(*F*_o_^2^) + (0.0626*P*)^2^ + 0.2118*P*] where *P* = (*F*_o_^2^ + 2*F*_c_^2^)/3
*wR*(*F*^2^) = 0.111	(Δ/σ)_max_ < 0.001
*S* = 1.06	Δρ_max_ = 0.23 e Å^−^^3^
2455 reflections	Δρ_min_ = −0.27 e Å^−^^3^
186 parameters	Extinction correction: *SHELXL2012* (Sheldrick, 2008), Fc^*^=kFc[1+0.001xFc^2^λ^3^/sin(2θ)]^-1/4^
0 restraints	Extinction coefficient: 0.0012 (3)
Primary atom site location: structure-invariant direct methods	

### Special details

**Table:**

Geometry. All esds (except the esd in the dihedral angle between two l.s. planes) are estimated using the full covariance matrix. The cell esds are taken into account individually in the estimation of esds in distances, angles and torsion angles; correlations between esds in cell parameters are only used when they are defined by crystal symmetry. An approximate (isotropic) treatment of cell esds is used for estimating esds involving l.s. planes.

### Fractional atomic coordinates and isotropic or equivalent isotropic displacement parameters (Å^2^)

**Table:**

	*x*	*y*	*z*	*U*_iso_*/*U*_eq_	
N1A	0.73318 (9)	0.69373 (17)	0.20819 (8)	0.0218 (3)	
H1AA	0.6882	0.7451	0.1554	0.026*	
H1AB	0.6968	0.5788	0.2299	0.026*	
N2A	0.91562 (9)	0.69808 (17)	0.33676 (8)	0.0212 (3)	
N3A	0.98791 (10)	0.77052 (18)	0.48251 (9)	0.0244 (3)	
N4A	1.09665 (9)	0.62261 (18)	0.37363 (9)	0.0248 (3)	
C1A	0.74267 (11)	0.8382 (2)	0.28219 (10)	0.0223 (3)	
H1AC	0.6694	0.8739	0.2997	0.027*	
H1AD	0.7785	0.9539	0.2605	0.027*	
C2A	0.80895 (11)	0.7569 (2)	0.36402 (10)	0.0214 (3)	
H2AA	0.8179	0.8540	0.4127	0.026*	
H2AB	0.7707	0.6463	0.3882	0.026*	
C3A	0.91155 (11)	0.5639 (2)	0.26058 (9)	0.0230 (3)	
H3AA	0.8806	0.4418	0.2798	0.028*	
H3AB	0.9861	0.5395	0.2429	0.028*	
C4A	0.84275 (11)	0.6420 (2)	0.17953 (10)	0.0232 (3)	
H4AA	0.8782	0.7557	0.1554	0.028*	
H4AB	0.8355	0.5453	0.1306	0.028*	
C5A	1.00316 (11)	0.69522 (19)	0.40052 (10)	0.0186 (3)	
C6A	1.07633 (13)	0.7795 (2)	0.54032 (11)	0.0281 (4)	
H6A	1.0695	0.8347	0.5985	0.034*	
C7A	1.17713 (12)	0.7133 (2)	0.52036 (12)	0.0287 (4)	
H7A	1.2390	0.7224	0.5625	0.034*	
C8A	1.18203 (12)	0.6329 (2)	0.43523 (12)	0.0285 (4)	
H8A	1.2495	0.5823	0.4194	0.034*	
O1B	0.45437 (8)	0.79460 (16)	0.54884 (7)	0.0247 (3)	
O2B	0.62685 (8)	0.69817 (16)	0.54483 (7)	0.0274 (3)	
O3B	0.48860 (8)	0.68609 (15)	0.21591 (6)	0.0227 (3)	
O4B	0.33707 (8)	0.85812 (15)	0.22066 (6)	0.0228 (3)	
C1B	0.53605 (11)	0.73177 (19)	0.50805 (9)	0.0170 (3)	
C2B	0.51599 (11)	0.70263 (19)	0.40767 (9)	0.0173 (3)	
H2B	0.5605	0.6157	0.3779	0.021*	
C3B	0.43843 (11)	0.79386 (19)	0.35918 (9)	0.0174 (3)	
H3B	0.3918	0.8740	0.3906	0.021*	
C4B	0.41910 (10)	0.77939 (19)	0.25776 (9)	0.0159 (3)	
H1B	0.473 (2)	0.801 (4)	0.624 (2)	0.091 (10)*	

### Atomic displacement parameters (Å^2^)

**Table:**

	*U*^11^	*U*^22^	*U*^33^	*U*^12^	*U*^13^	*U*^23^
N1A	0.0163 (6)	0.0294 (7)	0.0188 (6)	−0.0046 (5)	−0.0057 (5)	0.0051 (5)
N2A	0.0132 (6)	0.0313 (7)	0.0186 (6)	0.0027 (4)	−0.0023 (4)	−0.0025 (5)
N3A	0.0187 (6)	0.0321 (7)	0.0216 (7)	0.0000 (5)	−0.0037 (5)	−0.0027 (5)
N4A	0.0156 (6)	0.0291 (7)	0.0294 (7)	0.0033 (5)	−0.0019 (5)	−0.0006 (5)
C1A	0.0141 (6)	0.0272 (7)	0.0249 (7)	0.0001 (5)	−0.0025 (5)	0.0018 (6)
C2A	0.0128 (6)	0.0314 (7)	0.0194 (7)	0.0014 (5)	−0.0014 (5)	−0.0016 (6)
C3A	0.0208 (7)	0.0297 (8)	0.0180 (7)	0.0028 (5)	−0.0019 (5)	−0.0023 (6)
C4A	0.0214 (7)	0.0312 (8)	0.0165 (7)	−0.0020 (6)	−0.0009 (5)	0.0003 (6)
C5A	0.0145 (6)	0.0202 (7)	0.0204 (7)	−0.0013 (5)	−0.0031 (5)	0.0030 (5)
C6A	0.0260 (8)	0.0332 (8)	0.0236 (8)	−0.0031 (6)	−0.0082 (6)	−0.0005 (6)
C7A	0.0209 (7)	0.0297 (8)	0.0333 (9)	−0.0019 (6)	−0.0125 (6)	0.0050 (6)
C8A	0.0149 (7)	0.0286 (8)	0.0409 (9)	0.0023 (5)	−0.0054 (6)	0.0016 (7)
O1B	0.0196 (5)	0.0454 (7)	0.0092 (5)	0.0021 (4)	0.0008 (4)	−0.0006 (4)
O2B	0.0216 (5)	0.0433 (7)	0.0163 (5)	0.0075 (4)	−0.0064 (4)	−0.0044 (4)
O3B	0.0221 (5)	0.0362 (6)	0.0094 (5)	0.0091 (4)	−0.0006 (4)	−0.0012 (4)
O4B	0.0183 (5)	0.0329 (6)	0.0164 (5)	0.0056 (4)	−0.0043 (4)	−0.0029 (4)
C1B	0.0173 (6)	0.0209 (7)	0.0126 (7)	−0.0017 (5)	−0.0007 (5)	0.0011 (5)
C2B	0.0166 (6)	0.0241 (7)	0.0111 (6)	−0.0008 (5)	0.0009 (5)	−0.0004 (5)
C3B	0.0170 (6)	0.0244 (7)	0.0109 (6)	0.0008 (5)	0.0020 (5)	−0.0020 (5)
C4B	0.0153 (6)	0.0206 (7)	0.0115 (6)	−0.0012 (5)	−0.0001 (5)	0.0001 (5)

### Geometric parameters (Å, º)

**Table:**

N1A—H1AA	0.9900	C3A—C4A	1.5157 (19)
N1A—H1AB	0.9900	C4A—H4AA	0.9900
N1A—C1A	1.4883 (19)	C4A—H4AB	0.9900
N1A—C4A	1.4909 (18)	C6A—H6A	0.9500
N2A—C2A	1.4640 (17)	C6A—C7A	1.380 (2)
N2A—C3A	1.4637 (18)	C7A—H7A	0.9500
N2A—C5A	1.3782 (17)	C7A—C8A	1.380 (2)
N3A—C5A	1.3438 (19)	C8A—H8A	0.9500
N3A—C6A	1.3351 (19)	O1B—C1B	1.2890 (17)
N4A—C5A	1.3477 (18)	O1B—H1B	1.12 (3)
N4A—C8A	1.3407 (19)	O2B—C1B	1.2311 (17)
C1A—H1AC	0.9900	O3B—C4B	1.2742 (16)
C1A—H1AD	0.9900	O4B—C4B	1.2445 (16)
C1A—C2A	1.5173 (19)	C1B—C2B	1.4941 (17)
C2A—H2AA	0.9900	C2B—H2B	0.9500
C2A—H2AB	0.9900	C2B—C3B	1.3181 (19)
C3A—H3AA	0.9900	C3B—H3B	0.9500
C3A—H3AB	0.9900	C3B—C4B	1.4977 (17)
			
H1AA—N1A—H1AB	108.1	N1A—C4A—H4AA	109.8
C1A—N1A—H1AA	109.6	N1A—C4A—H4AB	109.8
C1A—N1A—H1AB	109.6	C3A—C4A—H4AA	109.8
C1A—N1A—C4A	110.45 (10)	C3A—C4A—H4AB	109.8
C4A—N1A—H1AA	109.6	H4AA—C4A—H4AB	108.2
C4A—N1A—H1AB	109.6	N3A—C5A—N2A	116.82 (12)
C3A—N2A—C2A	114.29 (11)	N3A—C5A—N4A	126.34 (13)
C5A—N2A—C2A	119.56 (12)	N4A—C5A—N2A	116.80 (13)
C5A—N2A—C3A	119.55 (11)	N3A—C6A—H6A	118.1
C6A—N3A—C5A	115.41 (13)	N3A—C6A—C7A	123.76 (15)
C8A—N4A—C5A	115.36 (13)	C7A—C6A—H6A	118.1
N1A—C1A—H1AC	109.8	C6A—C7A—H7A	122.2
N1A—C1A—H1AD	109.8	C8A—C7A—C6A	115.59 (14)
N1A—C1A—C2A	109.39 (11)	C8A—C7A—H7A	122.2
H1AC—C1A—H1AD	108.2	N4A—C8A—C7A	123.47 (14)
C2A—C1A—H1AC	109.8	N4A—C8A—H8A	118.3
C2A—C1A—H1AD	109.8	C7A—C8A—H8A	118.3
N2A—C2A—C1A	109.41 (12)	C1B—O1B—H1B	111.6 (15)
N2A—C2A—H2AA	109.8	O1B—C1B—C2B	115.42 (12)
N2A—C2A—H2AB	109.8	O2B—C1B—O1B	125.37 (12)
C1A—C2A—H2AA	109.8	O2B—C1B—C2B	119.20 (12)
C1A—C2A—H2AB	109.8	C1B—C2B—H2B	119.0
H2AA—C2A—H2AB	108.2	C3B—C2B—C1B	121.98 (13)
N2A—C3A—H3AA	109.5	C3B—C2B—H2B	119.0
N2A—C3A—H3AB	109.5	C2B—C3B—H3B	117.9
N2A—C3A—C4A	110.78 (12)	C2B—C3B—C4B	124.24 (12)
H3AA—C3A—H3AB	108.1	C4B—C3B—H3B	117.9
C4A—C3A—H3AA	109.5	O3B—C4B—C3B	116.94 (11)
C4A—C3A—H3AB	109.5	O4B—C4B—O3B	124.90 (12)
N1A—C4A—C3A	109.46 (11)	O4B—C4B—C3B	118.15 (12)
			
N1A—C1A—C2A—N2A	57.34 (15)	C5A—N3A—C6A—C7A	−1.5 (2)
N2A—C3A—C4A—N1A	−54.53 (16)	C5A—N4A—C8A—C7A	−0.6 (2)
N3A—C6A—C7A—C8A	−0.6 (2)	C6A—N3A—C5A—N2A	−174.86 (13)
C1A—N1A—C4A—C3A	59.07 (15)	C6A—N3A—C5A—N4A	2.9 (2)
C2A—N2A—C3A—C4A	54.41 (16)	C6A—C7A—C8A—N4A	1.8 (2)
C2A—N2A—C5A—N3A	−8.22 (19)	C8A—N4A—C5A—N2A	175.90 (13)
C2A—N2A—C5A—N4A	173.80 (12)	C8A—N4A—C5A—N3A	−1.9 (2)
C3A—N2A—C2A—C1A	−55.43 (16)	O1B—C1B—C2B—C3B	23.25 (19)
C3A—N2A—C5A—N3A	−158.13 (13)	O2B—C1B—C2B—C3B	−155.66 (14)
C3A—N2A—C5A—N4A	23.89 (19)	C1B—C2B—C3B—C4B	176.05 (12)
C4A—N1A—C1A—C2A	−60.83 (14)	C2B—C3B—C4B—O3B	−6.6 (2)
C5A—N2A—C2A—C1A	153.15 (13)	C2B—C3B—C4B—O4B	173.29 (13)
C5A—N2A—C3A—C4A	−154.17 (13)		

### Hydrogen-bond geometry (Å, º)

**Table:**

*D*—H···*A*	*D*—H	H···*A*	*D*···*A*	*D*—H···*A*
N1*A*—H1*AA*···O2*B*^i^	0.99	1.79	2.7601 (15)	166
N1*A*—H1*AB*···O4*B*^ii^	0.99	1.78	2.7493 (16)	167
C8*A*—H8*A*···O2*B*^iii^	0.95	2.53	3.3133 (18)	140
O1*B*—H1*B*···O3*B*^iv^	1.12 (3)	1.35 (3)	2.4679 (13)	176 (3)

Symmetry codes: (i) *x*, −*y*+3/2, *z*−1/2; (ii) −*x*+1, *y*−1/2, −*z*+1/2; (iii) −*x*+2, −*y*+1, −*z*+1; (iv) *x*, −*y*+3/2, *z*+1/2.
